# Progress in State Medicine

**Published:** 1900-08-18

**Authors:** 


### PROGRESS IN STATE MEDICINE.

**Sewage Disposal.**[1]—Bacteriological methods of sewage disposal may be aërobic or anaërobic, and in either case by the agency of living micro-organisms complex organic bodies are resolved into those of a simpler nature. Bacteria do not thrive well in acid media, so that in large towns where there is much trade refuse (waste from chemica works, &c.), let into the sewers, especially at fixed times instead of being spread over the 24 hours, the aërobic system would not work well. But even under most satisfactory conditions the effluent is not free from bacteria; and, although pathogenic organisms are rare in crude sewage, should the outfall be into a river from which human beings may drink, some sort of filter may be interposed. There is difficulty in preventing the coarser part of the sewage, such as straw, rags, and paper, from finding its way on to the beds, and so clogging them. The most satisfactory method to prevent this is to pass the sewage over a "flume," or open box, which is divided into transverse partitions, and which is slightly sloped down to the filter-bed, thus allowing the heavier particles to sink into the transverse partitions.

Stoddart[2] describes a new form of sewage filter of his own construction. It is best described under three heads: (α) supply channel; (β) distributor; and (γ) filter. The supply channel consists of a gutter in direct communication with the tank outlet. Its floor is gently sloped to one end, and here a valve may be placed for flushing out any deposit. A baffle plate is situated near the inlet to regulate the rate of flow. The sewage overflowing the edges of the gutter runs into the patent distributor (β). This is a number of narrow gutters placed at right angles to the supply channel. Along the lowest part of each gutter underneath are arranged a series of vertical points. Thus the sewage overflowing the supply channel runs into the distributor, overflows these smaller gutters, and running down their under surface falls from the vertical points in small drops into the filter (γ). The filter is composed of a heap of coarse rubble, clinker, coke, and ballast placed on a sloped concrete floor, which enables any liquid reaching it to flow to the nearest external surface. The filtrate is collected by an external channel surrounding the filter. As there is at no time any body of water collected in the filter, a retaining wall is said not to be needed except in sma l installations. Neither severe frost or heavy rainfall have interfered with its working. The filter matures rapidly. At the end of the second month the effluent is perfectly clear, and is not only capable of admission into any watercourse without risk of offence, but is in a condition to effect a marked improvement in polluted water into which it may be discharged. A filter of this description has been working night and day for a considerable time at Bristol, and up to the present has given excellent results.

**Septic Tank.**—The Special Commissioner[3] of the *British Medical Journal* gives the following description of the septic tank process. It consists of a chamber built below the level of the ground of cemented masonry. This chamber is in direct communication with the sewers, and is covered over by a layer of soil and turf, thus making it practically air and light tight. To prevent heavy masses passing into this, a deeper tank, known as the "grit chamber" is interposed between it and the sewer. It is so arranged that the grit chamber can be easily cleaned out without disturbing the main tank itself. The sewage enters by two inlets, without any screening, and is carried down to a depth of five feet below the surface. This prevents the disturbance of the scum on the surface of the sewage, excludes all air, and prevents the escape of gases back into the sewer. The flow is continuous. The influent and effluent are on the same level, situated about half way between the surface of the sewage and the deposit at the bottom of the tank. In addition a cast-iron pipe crossing the whole width of the tank, placed about fifteen inches below the surface, provides another effluent through a slot in its under surface. Thus no currents are formed, and there is very little disturbance to the scum on the surface or of the finer deposit at the bottom, and plenty of time is allowed for the action of the bacteria, owing to the tank itself holding almost exactly one day's average flow. It takes some time for the bacteria to be in such a condition as to grapple with the sewage, but this once accomplished no further attention is needed in this respect. An inspection-chamber enables the progress in the septic tank to be watched. The deposit is small, and can be pumped out. The effluent, now of a yellowish-brown colour and with a distinct odour, flows through a pipe into an open chamber outside the tank. From here it runs into a trough closed at both ends. Overflowing, it runs down into another grooved channel, thus getting partially oxidised. From here it is conducted into two valve chambers, and from them to the filters. These are made of crushed furnace clinker resting on six inches of gravel, and are about four and a half feet deep. Owing to the limited amount of oxygen available in these filters it is essential that they should be used intermittently. The effluent is clear and bright, and has an almost imperceptible smell. Gases are generated in the tank; and at Exeter, where the septic tank is in operation, the hydrogen and marsh gas with the aid of an incandescent mantle are made to provide light to illuminate the works at night. Storm water has little effect on the rate of flow in the tank. Pathogenic water-borne organisms are generally supposed to be destroyed in the tanks. The initial cost of erection of this system is small, and the subsequent outlay equally so. At present the septic tank system seems most suitable to small towns, villages, or large country establishments.

The septic tank[4] system has been an apparent failure in Leicester, due probably to the fact that the sewage had already undergone excessive anaërobic action in the sewers. Numerous experiments were tried, but the main result deduced was that the final effluent was much more satisfactory where simple filter beds and land irrigation were employed than when the sewage was passed into a septic tank. Faulty sewers, low gradients, or any cause tending to prevent a free flow from the sewers to the tank would militate against a proper bacteriological action taking place in the sewage when it arrived at the septic chamber. However, the experiments certainly do point, as far as Leicester is concerned, to the fact that the septic tank is not a necessity in dealing with the town's sewage.

[1] Brit. Med. Jour., Jan. 6, 1900. [2] Public Health Engineer, 1900. [3] Brit. Med. Jour., Jan. 13, 1900. [4] Ibid., July 21.

